# Sorafenib suppresses extrahepatic metastasis *de novo* in hepatocellular carcinoma through inhibition of mesenchymal cancer stem cells characterized by the expression of CD90

**DOI:** 10.1038/s41598-017-11848-z

**Published:** 2017-09-12

**Authors:** Mariko Yoshida, Taro Yamashita, Hikari Okada, Naoki Oishi, Kouki Nio, Takehiro Hayashi, Yoshimoto Nomura, Tomoyuki Hayashi, Yoshiro Asahina, Mika Ohwada, Hajime Sunagozaka, Hajime Takatori, Federico Colombo, Laura Porretti, Masao Honda, Shuichi Kaneko

**Affiliations:** 1 Department of Gastroenterology, Kanazawa University Graduate School of Medical Science, Kanazawa, Ishikawa 920-8641 Japan; 2 Department of General Medicine, Kanazawa University Hospital, Kanazawa, Ishikawa 920-8641 Japan; 3 Clinical Chemistry and Microbiology Laboratory, Flow Cytometry and Experimental Hepatology Service, Fondazione IRCCS Ca’ Granda Ospedale Maggiore Policlinico, Milan, Italy

## Abstract

Cancer stem cells (CSCs) are a pivotal target for eradicating hepatocellular carcinoma (HCC). We previously reported that distinctive CSCs regulating tumorigenicity (EpCAM^+^ CSCs) and metastasis (CD90^+^ CSCs) have different epithelial/mesenchymal gene expression signatures. Here, we examined the influence of sorafenib, a multiple-receptor tyrosine kinase inhibitor used as a first-line treatment for advanced HCC, on EpCAM^+^ and CD90^+^ CSCs. CD90^+^ cells showed higher c-Kit gene/protein expression than EpCAM^+^ cells. Sorafenib treatment reduced the number of CD90^+^ cells with attenuated c-Kit phosphorylation, whereas it enriched the EpCAM^+^ cell population. We evaluated the role of CD90^+^ and EpCAM^+^ CSCs *in vivo* by subcutaneously injecting these CSCs together in immune-deficient mice. We observed that sorafenib subtly affected the suppression of primary tumor growth maintained by EpCAM^+^ CSCs, but completely inhibited the lung metastasis mediated by CD90^+^ CSCs. We further evaluated the effect of sorafenib on extracellular vesicle (EV) production and found that sorafenib suppressed the production of EVs containing TGF-β mRNA in CD90^+^ cells and inhibited the cell-cell communication and motility of EpCAM^+^ cells. Our data suggest the following novel effects of sorafenib: suppressing CD90^+^ CSCs and inhibiting the production of EVs regulating distant metastasis.

## Introduction

While considered monoclonal in origin, cancer is a heterogeneous disease in terms of morphology, biological behavior, chemo/radiation resistance, and prognosis. Traditionally, this heterogeneity has been attributed to the clonal evolution of tumor cells with the stochastic accumulation of genetic/epigenetic/genomic changes^1^. However, recent studies have suggested that cancer cell heterogeneity can also be explained by the hierarchical organization of the tumor mediated by a subset of cells with stem/progenitor cell features called cancer stem cells (CSCs)^2^. As normal stem cells can repopulate the cell lineages of the corresponding organ, CSCs can divide symmetrically (self-renewal capacity) and asymmetrically (differentiation capacity) to repopulate the tumor^3^. CSCs generally express normal stem/progenitor cell markers, are highly tumorigenic/metastatic, and show chemo/radiation resistance. Therefore, the eradication of CSCs is considered pivotal in the treatment of cancer.

Hepatocellular carcinoma (HCC) is a leading cause of cancer death worldwide. Recent evidence has proven that HCC is also driven by CSCs expressing various hepatic stem/progenitor markers such as EpCAM, CD133, CD90, and CD44^4^. We previously demonstrated that EpCAM^+^ HCC cells isolated from primary HCC and cell lines showed CSC features including tumorigenicity, invasiveness, and resistance to fluorouracil^5, 6^. We further found that EpCAM^+^ cells and CD90^+^ cells exist distinctively in primary HCCs with unique gene and protein expression profiles. We found that EpCAM^+^ CSCs showed highly tumorigenic capacity with the expression of classical hepatic stem/progenitor cell lineage markers such as *KRT19* and *AFP*. In contrast, although the function of CD90 is still under debate, we revealed that CD90^+^ CSCs showed high metastatic capacity with the expression of mesenchymal stem cell markers such as *KIT* and *FLT1* ^7^. These data suggest that CSCs are not a single entity and that distinct EpCAM^+^ and CD90^+^ CSCs could collaborate to orchestrate tumor progression and metastasis, suggesting that both EpCAM^+^ and CD90^+^ CSCs should be eradicated in the treatment of HCC^8^.

As CSCs are considered resistant to classical cytotoxic agents such as 5-fluorouracil and epirubicin, efforts have been made to evaluate the effects of molecularly targeted agents on CSCs. We previously found that EpCAM^+^ CSCs showed activation of the transcription factor SALL4 and chromatin remodeling enzyme chromodomain-helicase-DNA-binding protein 4 (CHD4), resulting in the recruitment of the nucleosome remodeling and histone deacetylase (NuRD) complex at certain genome regions^9^. Activation of the NuRD complex is associated with high histone deacetylase (HDAC) activity and DNA double strand break repair mediated by poly (ADP-ribose) polymerase (PARP), which is closely related to the stemness and chemoresistance of EpCAM^+^ CSCs^10^. Notably, molecular inhibition of the NuRD complex with an HDAC inhibitor and a PARP inhibitor successfully eradicated tumorigenic EpCAM^+^ CSCs. However, these agents had limited effects on the eradication of metastatic CD90^+^ CSCs, warranting further efforts to identify potential molecularly targeted agents that can effectively eradicate metastatic mesenchymal CSCs^11^.

In this study, we evaluated the effects of sorafenib, a multiple receptor tyrosine kinase inhibitor and the first molecularly targeted anticancer agent proven to prolong overall survival in patients with advanced HCC. We found that sorafenib can target CD90^+^ metastatic CSCs potentially through inhibition of c-Kit signaling and suppress the extrahepatic metastasis of HCC *in vitro* and *in vivo*.

## Results

### Activation of c-Kit signaling in mesenchymal CD90^+^ CSCs

**Figure 1:**
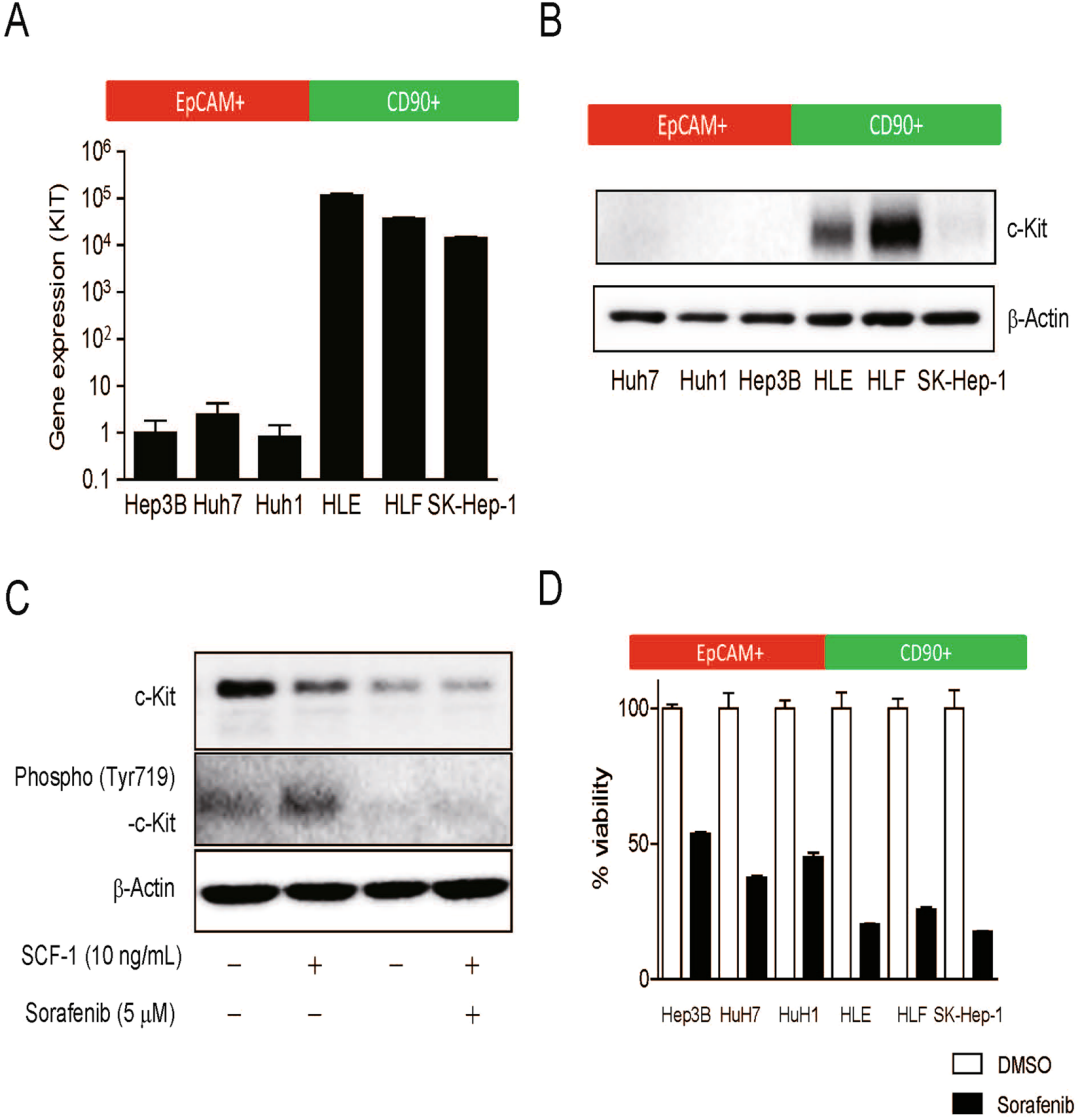
Differential activation of c-Kit signaling in EpCAM^+^ and CD90^+^ HCC cell lines. (**A**) qRT-PCR analysis of EpCAM^+^ (Hep3B, HuH7, and HuH1) and CD90^+^ (HLE, HLF, SK-Hep-1) HCC cell lines. (**B**) Western blot analysis of c-Kit expression in EpCAM^+^ (Hep3B, HuH7, and HuH1) and CD90^+^ (HLE, HLF, SK-Hep-1) HCC cell lines. (**C**) Western blot analysis of c-Kit and phospho-c-Kit in HLF cells treated with SCF-1 and sorafenib for 24 h. (**D**) Cell proliferation assay of EpCAM^+^ (Hep3B, HuH7, and HuH1) and CD90^+^ (HLE, HLF, SK-Hep-1) HCC cell lines treated with vehicle (0.1% DMSO) or sorafenib (5 μM).

We first explored the expression of the mesenchymal stem cell marker KIT in six representative HCC cell lines that we had evaluated previously (Hep3B, HuH7, and HuH1 as EpCAM^+^ cell lines, and HLE, HLF, and SK-Hep-1 as CD90^+^ cell lines). We also performed co-culture experiment of EpCAM^+^ Huh7 cells and CD90^+^ HLF cells by time-lapse image analysis (Supplementary movie 1). Interestingly, although EpCAM^+^ and CD90^+^ cells were mixed well to equally disperse in the well, EpCAM^+^ cells autonomously generated the epithelial nodule-like structure. In contrast, CD90^+^ cells autonomously formed stroma-like structure surrounding the nodule formed by EpCAM^+^ cells, suggesting the different natures of epithelial EpCAM^+^ and mesenchymal CD90^+^ cell lines. We evaluated the role of EpCAM and CD90 expression in HCC cell lines (Supplementary Fig. 1). Knockdown of EpCAM (encoded by EPCAM) or CD90 (encoded by Thy1) was confirmed by quantitative reverse-transcription polymerase chain reaction (qRT-PCR) analysis, and EpCAM knockdown slightly suppressed the proliferation of HuH7 cells, consistent with previous studies on the role of EpCAM in Wnt signaling activation and stemness^5, 12^. In contrast, CD90 knockdown had no effect on the proliferation of HLF cells. We found that the CD90^+^ HCC cell lines expressed KIT abundantly compared with the EpCAM^+^ HCC cell lines, and the difference in gene expression was approximately 4-5 log, as evaluated by qRT-PCR analysis (Fig. 1A). We further evaluated the expression of c-Kit protein in these cell lines by Western blotting. c-Kit was strongly expressed in the CD90^+^ HLE and HLF cell lines and weakly expressed in SK-Hep-1 cells, while its expression was negligible in the EpCAM^+^ HCC cell lines (Fig. 1B). We investigated whether c-Kit signaling was activated by its ligand stem cell factor 1 (SCF-1) in HLF cells. Phosphorylation of c-Kit was increased in the presence of SCF-1 (10 ng/mL) for 24 h compared with the vehicle-treated (control) group, and sorafenib treatment (5 μM) dramatically suppressed the phosphorylation of c-Kit either with or without SCF-1 treatment (Fig. 1C). We evaluated the effect of sorafenib on cell proliferation in the EpCAM^+^ and CD90^+^ HCC cell lines; sorafenib treatment (10 μM) suppressed the proliferation of the CD90^+^ HCC cell lines (HLE, HLF, and SK-Hep-1) more strongly compared with the EpCAM^+^ HCC cell lines (HuH1, HuH7, and Hep3B) and this was statistically significant (P < 0.05) (Fig. 1D). These data suggest that sorafenib inhibited c-Kit signaling and suppressed the proliferation of the CD90^+^ HCC cell lines more effectively than in the EpCAM^+^ HCC cell lines.

### Differential chemosensitivity to sorafenib in HCC clones obtained from the same ancestor with distinct EpCAM/CD90 expression status

**Figure 2:**
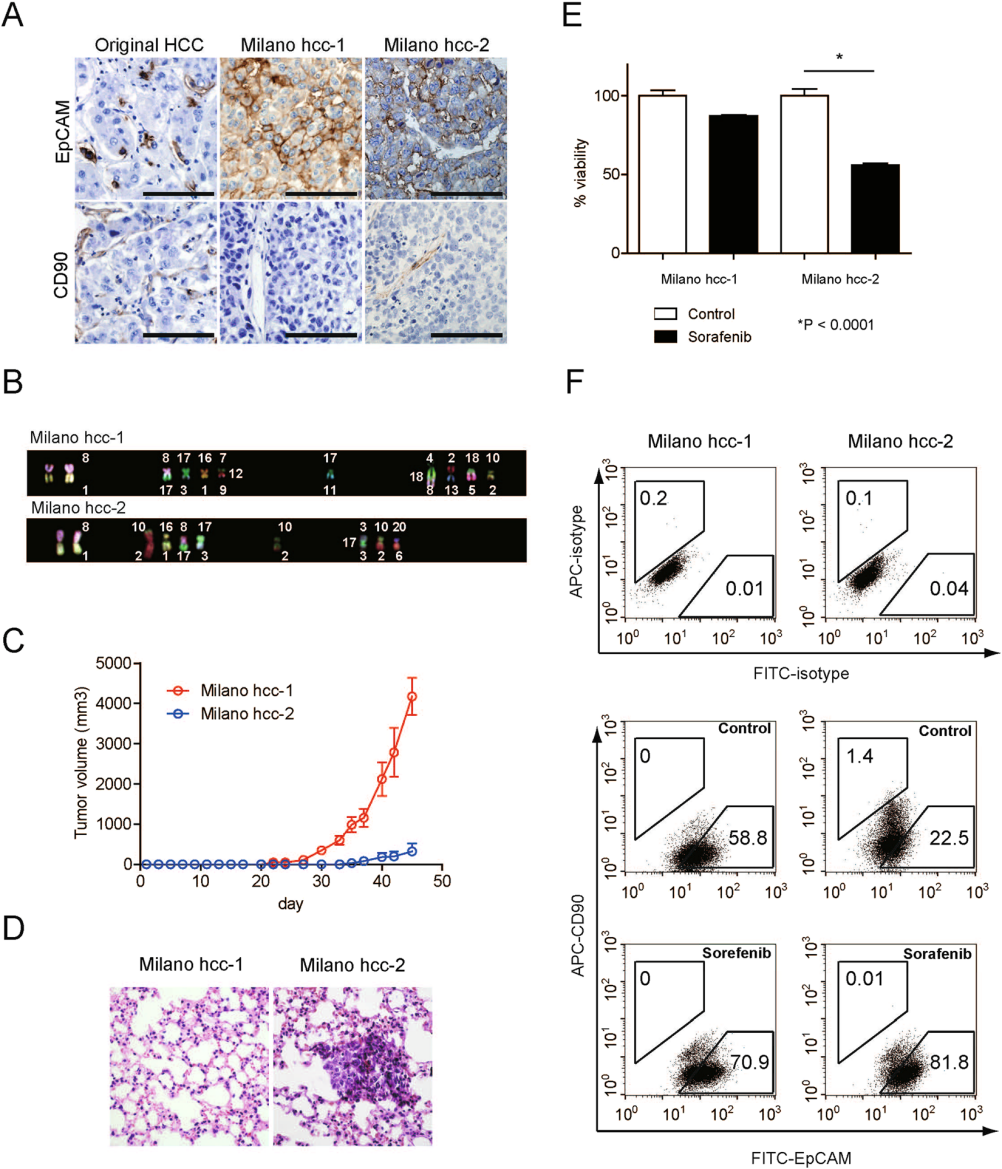
Characteristics of two distinct HCC clones derived from the same ancestor. (**A**) Immunohistochemical analysis of original HCC tissue and Milano hcc-1 and -2 tumor tissues developed in NOD/SCID xenotransplant mice. (**B**) Multicolor FISH analysis of Milano hcc-1 and -2 clones. (**C**) Tumorigenic capacity of Milano hcc-1 and -2 clones injected subcutaneously in the flank of NOD/SCID mice. (**D**) Microscopic evaluation of metastasis in the lung of NOD/SCID mice. (**E**) Cell proliferation assay of Milano hcc-1 and -2 clones treated with vehicle (control) or sorafenib (2.5 μM) for 72 h. (**F**) FACS analysis of EpCAM and CD90 expression in Milano hcc-1 and -2 clones treated with control (0.1%) or sorafenib (5 μM) for 72 h.

Primary HCC tissues are known to be composed of a variety of EpCAM^+/−^ and CD90^+/−^ cancer cells. As the representative HCC cell lines we investigated were established from samples obtained from different patients, it is possible that the difference in chemosensitivity of the EpCAM^+^ and CD90^+^ HCC cells to sorafenib treatment may be related to the different genetic/epigenetic/genomic changes that these cell lines have acquired independently (intertumor heterogeneity). To evaluate the sensitivity of HCC cells with distinct EpCAM/CD90 expression status to sorafenib, we utilized two HCC clones (Milano hcc-1 and Milano hcc-2) derived from a previously established single HCC tissue (intratumor heterogeneity)^13^. Histologically, the original HCC sample showed EpCAM expression in tumor epithelial cells and CD90 expression in mesenchymal cells (Fig. 2A). Notably, subcutaneous tumor tissues obtained from Milano hcc-2 contained similar epithelial cell-shaped EpCAM^+^ cells and mesenchymal cell-shaped CD90^+^ cells (Fig. 2A). In contrast, tumor tissue established by the Milano hcc-1 clone did not contain CD90^+^ cells, even in vascular areas. Multicolor fluorescent *in-situ* hybridization (FISH) analysis indicated that Milano hcc-1 and hcc-2 shared common chromosomal alterations (chromosome 1:8 fusion) (Fig. 2B). We isolated CD90^+^ or EpCAM^+^ cells from Milano hcc-2 cells by cell sorting, and found that EpCAM^+^ cells could repopulate the original CD90^+^ or EpCAM^−^ CD90^−^ cell population within 30 days (Supplementary Fig. 2A). In contrast, CD90^+^ cells could generate EpCAM^−^ CD90^−^ cells, but rarely generated EpCAM^+^ cells, suggesting that EpCAM^+^ cells are CSCs that can generate CD90^+^ progenitors and EpCAM^−^ CD90^−^ cells, at least in Milano hcc-2 cells. The high tumorigenic capacity of sorted EpCAM^+^ cells compared with unsorted cells was confirmed *in vivo*, although the difference did not reach statistical significance (Supplementary Fig. 2B and C). We evaluated tumorigenic capacity and metastatic capacity by using the Milano hcc-1 and -2 clones. Interestingly, Milano hcc-1, which showed more chromosomal abnormalities with the loss of the CD90^+^ cell population, demonstrated a higher tumorigenic capacity than Milano hcc-2 (Fig. 2C). In contrast, lung metastasis was detected for only Milano hcc-2, which contained CD90^+^ cells, after subcutaneous injection (Fig. 2D). Despite showing the highest tumorigenic capacity, Milano hcc-1 did not metastasize to the lungs in a subcutaneous transplantation model. These data suggested that although EpCAM^+^ cells are CSCs, if they did not generate CD90^+^ progenitors with mesenchymal cell features, they could not metastasize to the distant organ, at least in Milano hcc cells. When we evaluated the chemosensitivity of the two clones to sorafenib, we found that Milano hcc-2, containing a small population of CD90^+^ cells, demonstrated significant chemosensitivity to the drug (P < 0.0001) (Fig. 2E). In contrast, Milano hcc-1 cells, containing only EpCAM^+^ cells, did not show such chemosensitivity. Fluorescence-activated cell sorting (FACS) analysis demonstrated that the population of CD90^+^ cells was maintained in the Milano hcc-2 clone, whereas this population was lost in the Milano hcc-1 clone (Fig. 2F). Sorafenib treatment resulted in the loss of CD90^+^ cells in Milano hcc-2, whereas EpCAM^+^ cells were enriched in both the Milano hcc-1 and -2 clones. These data highlight the fact that EpCAM^+^ and CD90^+^ cells can originate from the same ancestral cells, with distinct tumorigenic/metastatic capacities and chemosensitivity to certain molecularly targeted agents such as sorafenib.

### Suppression of mesenchymal CD90^+^ CSCs and inhibition of extrahepatic metastasis in HCC by sorafenib

**Figure 3:**
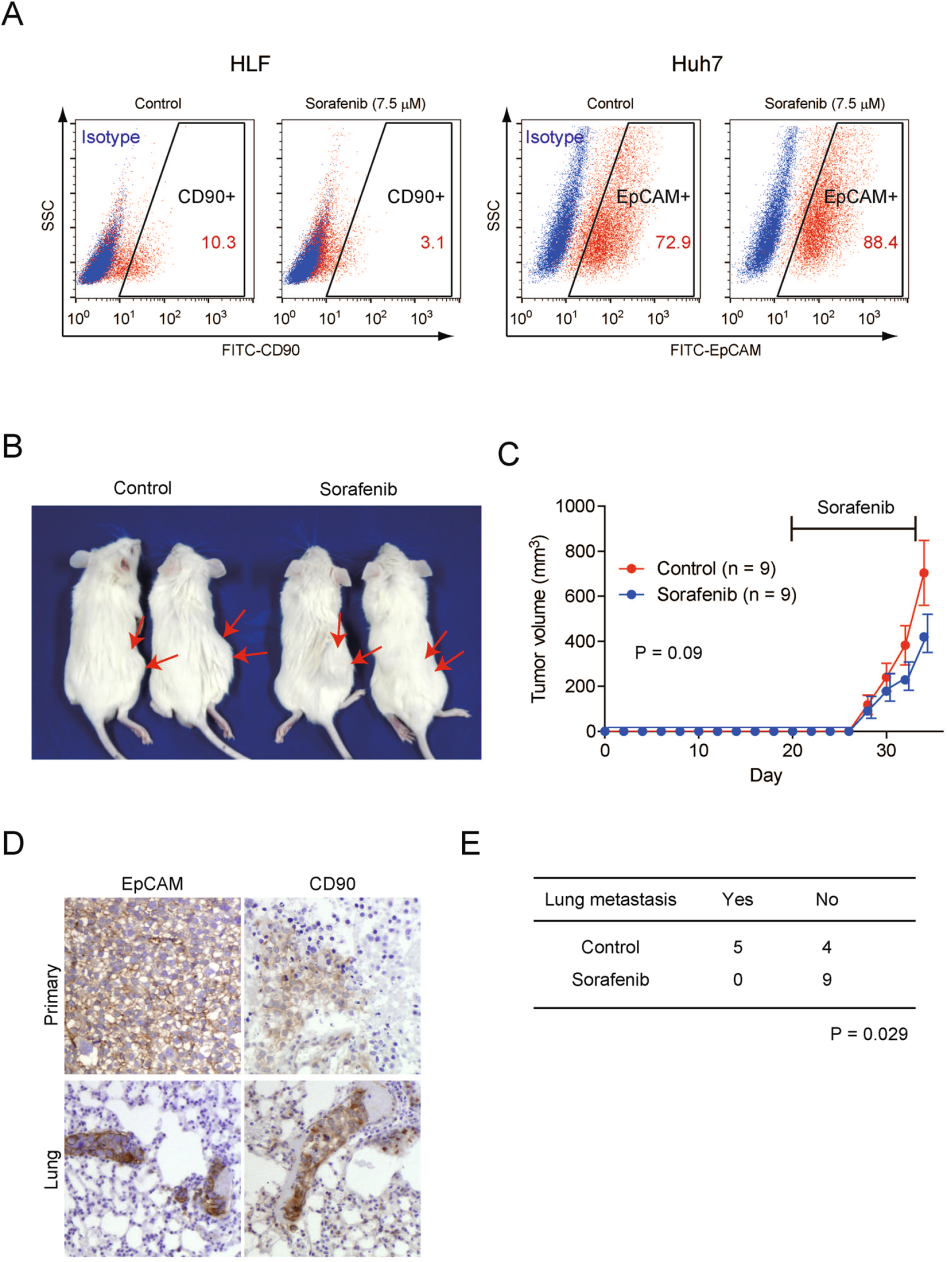
Sorafenib targets CD90^+^ HCC cells. (**A**) FACS analysis of CD90 and EpCAM expression in HLF and HuH7 cells treated with vehicle or sorafenib (7.5 μM) for 72 h. (**B**) Representative NOD/SCID mice with subcutaneous tumors from the combination of 5.0 × 10^5^ EpCAM^+^ HuH7 cells and 5.0 × 10^5^ CD90^+^ HLF cells treated with vehicle or sorafenib. (**C**) Tumorigenic capacity of 5.0 × 10^5^ EpCAM^+^ HuH7 cells and 5.0 × 10^5^ CD90^+^ HLF cells co-injected into a subcutaneous lesion and treated with vehicle or sorafenib. Sorafenib (30 mg/kg/day, 100 μL/mice, n = 9) or vehicle (100 μL/mice, n = 9) was orally administered 3 times per week at 20 days after injection for 2 weeks (day 20 to 34). (**D**) Immunohistochemical analysis of EpCAM and CD90 expression in primary tumors and lung metastasis. NOD/SCID mice treated with vehicle or sorafenib were sacrificed at day 34 and tissues were fixed with formalin. (**E**) Frequency of lung metastasis in NOD/SCID mice treated with vehicle (n = 9) or sorafenib (n = 9) for 2 weeks.

To explore the effect of sorafenib on the population of CD90^+^ CSCs, we treated HLF and HuH7 cells with sorafenib at a concentration of 7.5 μM for 72 h. We observed a dramatic reduction (from 10.3% to 3.1%) of CD90^+^ cells in the HLF cells (Fig. 3A). In contrast, sorafenib treatment enriched the EpCAM^+^ cell population (from 72.9% to 88.4%) in the HuH7 cells. We isolated EpCAM^+/−^ HuH7 cells and treated them with sorafenib, and found that the EpCAM^+^ cells were chemoresistant to sorafenib (Supplementary Fig. 3). These data suggest that sorafenib suppressed the metastatic CD90^+^ CSC population to inhibit *de novo* metastasis, but had a limited effect on the inhibition of the tumorigenic EpCAM^+^ CSC population, resulting in the growth of the primary tumor. We also evaluated the effect of EpCAM and CD90 knock down on sorafenib sensitivity in Huh7 and HLF cells. Surprisingly, CD90 knockdown resulted in the enhanced chemosensitivity to sorafenib in HLF cells (Supplementary Fig. 3B). In contrast, EpCAM knockdown had no such effect in Huh7 cells. Although the role of CD90 in cancer cell signaling is still under debate, our data suggested that CD90 may be a functional molecule to regulate sorafenib sensitivity in HCC. We utilized the HuH7 and HLF cells in a subcutaneous co-injection model, because this model uses EpCAM^+^ HuH7 cells (which originally show no metastatic capacity) and CD90^+^ HLF cells (which originally show weak tumorigenic capacity, but enhance the metastasis of HuH7 cells when they co-exist). Therefore, this model allowed us to evaluate the role of tumorigenic EpCAM^+^ CSCs and metastatic CD90^+^ CSCs at the same time by measuring the growth of the primary tumor and metastatic lung nodules. Compared with the control vehicle, sorafenib treatment (30 mg/kg, 3 times/week) inhibited primary tumor growth, although the difference did not reach statistical significance (P = 0.09, unpaired t-test) (Fig. 3B and C). We found that most of the primary tumor cells expressed EpCAM, whereas approximately 10% of cells expressed CD90 in control mice (Fig. 3D upper panels). We also found that EpCAM^+^ and CD90^+^ cells were almost equally detected in metastatic tumors (Fig. 3D lower panels), consistent with the pivotal role of CD90^+^ cells in metastasis. Noticeably, sorafenib treatment completely suppressed lung metastasis compared with the control, and the difference reached statistical significance (P = 0.029, Fisher’s exact test) (Fig. 3E). We further performed similar experiments using Milano hcc-2 cells, which originally contain both CD90^+^ and EpCAM^+^ cells (Supplemental Fig. 4). Sorafenib treatment modestly suppressed primary tumor growth without statistical significance (Supplementary Fig. 4A), but completely suppressed lung metastasis (Supplementary Fig. 4B and C). These data suggest that sorafenib could target the population of metastatic CD90^+^ CSCs, but had little effect on epithelial EpCAM^+^ CSCs in HCC.

### Suppression of extracellular vesicle secretion in CD90^+^ CSCs and inhibition of cellular communication between EpCAM^+^ and CD90^+^ CSCs by sorafenib

**Figure 4:**
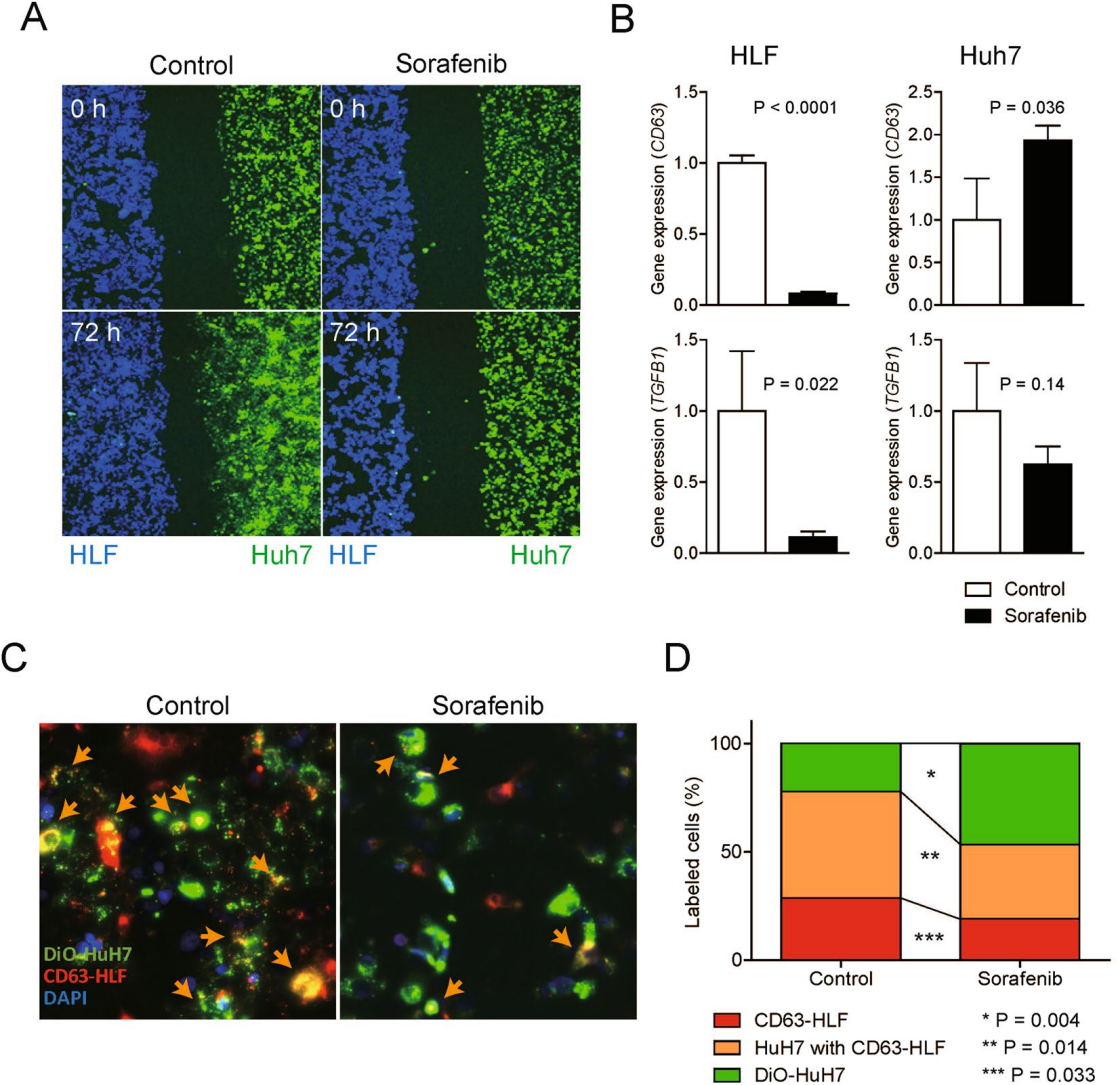
Sorafenib targets CD90^+^ cells to suppress EV secretion. (**A**) Cell motility of HuH7 cells (green) co-cultured with HLF cells (blue) with/without sorafenib was monitored in real-time by time-lapse image analysis. See also Supplementary Movies 2 and 3. (**B**) qRT-PCR analysis of *CD63* and *TGFB1* obtained from the EVs secreted from HLF and HuH7 cells treated with vehicle or sorafenib (2.5 μM). (**C**) Immunofluorescence analysis of HuH7 cells (green) cultured with CD63-labeled HLF cells (red). Sorafenib suppressed the number of HuH7 cells capturing EVs secreted from HLF cells (merge cells yellow). (**D**) Percentages of HuH7 cells (green), HLF cells (red), and HuH7 cells capturing EVs secreted from HLF cells (yellow). The number of green, red, and yellow cells was counted in triplicate at three independent areas. Sorafenib treatment significantly reduced the percentage of HuH7 cells trapping EVs produced by HLF cells.

The above data suggest that EpCAM^+^ CSCs and CD90^+^ CSCs may communicate with each other to determine the cancer phenotype in terms of tumorigenesis and metastasis of HCC. We performed a wound healing assay using HLF cells (labeled with DiD and indicated as blue) and HuH7 cells (labeled with DiO and indicated as green) by time-lapse image analysis (Fig. 4A). HuH7 cells showed enhanced cell motility in the presence of HLF cells, consistent with our previous data showing that HLF cells produce abundant TGF-β compared with HuH7 cells to enhance the motility of HuH7 in a paracrine manner (Supplementary Movie 2)^7^. Sorafenib treatment not only suppressed cell motility but also reduced the number of HLF cells. Furthermore, the enhanced motility of HuH7 cells induced by the presence of HLF cells was almost completely abolished by sorafenib treatment, without affecting the vibrant movements of HuH7 cells (Supplementary Movie 3). These data suggest that sorafenib not only suppressed the proliferation of HLF cells but also blocked cell-cell communication between CD90^+^ HLF and EpCAM^+^ HuH7 cells. We therefore investigated the status of extracellular vesicles (EVs) produced by HuH7 and HLF cells, because EVs are recognized as a pivotal molecular communication tool between neighboring or distant cells in the body^14^. Sorafenib treatment dramatically reduced the amount of EVs containing RNAs encoding *CD63* in HLF cells (Fig. 4B). In contrast, the same treatment increased the amount of EVs containing RNAs encoding *CD63* in HuH7 cells, suggesting that sorafenib differently affected the production of EVs between HLF and HuH7 cells. Furthermore, sorafenib treatment suppressed the number EVs containing RNAs encoding *TGFB1* in HLF cells, but had no such effect on HuH7 cells. We evaluated the status of the EVs produced by HLF cells (stained by PKH-26 and indicated in red) and co-cultured with HuH7 cells labeled with DiO (green) for 72 h (Fig. 4C). Approximately half of the HuH7 cells (49.2% ± 4.5%) trapped EVs produced by HLF cells (orange arrow) in the vehicle treatment group. Interestingly, sorafenib treatment significantly reduced the number of HuH7 cells trapping EVs (34.2% ± 4.2%) produced by HLF cells, and reduced the population of HLF cells (Fig. 4D), most likely due to the death of CD90^+^ cells. Taken together, all of these data suggest different effects of sorafenib on EpCAM^+^ and CD90^+^ HCC cells. In cancer cells, sorafenib mainly targets CD90^+^ CSCs to suppress the *de novo* metastasis of HCC, potentially through the suppression of EVs derived from CD90^+^ CSCs.

Most patients with advanced HCC who received sorafenib treatment rarely presented with the clinical response criteria outlined by the modified Response Evaluation Criteria in Solid Tumors for HCC^15–17^. Nevertheless, sorafenib treatment improved overall survival with statistical significance compared with placebo. Furthermore, other multiple-receptor tyrosine kinase inhibitors, such as brivanib, sunitinib, and axitinib, failed to prolong overall survival in patients with advanced HCC compared with sorafenib^18–20^. One possible reason why sorafenib was superior in prolonging overall survival might be its safety in patients with liver cirrhosis. However, recent data suggest that sorafenib also transiently reduced hepatic reserve^21^, suggesting that other potential mechanisms may exist to explain the superiority of sorafenib compared with other molecularly targeted agents. Sorafenib is considered to target mainly vascular endothelial cells to inhibit angiogenesis^22^, but our data suggest that sorafenib effectively inhibited CD90^+^ liver CSCs to suppress *de novo* metastasis. Although the mechanism is still under debate^23, 24^, our data suggest that the suppression of CD90^+^ liver CSCs may be mediated in part through the inhibition of c-Kit signaling.

We previously demonstrated that CSCs are heterogeneous in terms of tumorigenic and metastatic capacity in HCC^7^. EpCAM^+^ CSCs are highly tumorigenic and often invade the portal vein, but rarely metastasize to distant organs when isolated in pure culture and inoculated in immunodeficient mice. In contrast, CD90^+^ CSCs show mesenchymal cell morphology and regulate distant organ metastasis through the secretion of TGF-β. However, in the presence of CD90^+^ CSCs, we found that EpCAM^+^ CSCs, which originally showed no metastatic capacity, could metastasize to distant organs through the activation of the TGF-β/SMAD signaling pathway mediated by CD90^+^ CSCs^7, 8^. In this study, we further demonstrated that sorafenib attenuated c-Kit signaling in CD90^+^ cells *in vitro*, and suppressed distant organ metastasis with a limited effect on primary tumor growth *in vivo*. Besides, we found the enhanced chemosensitivity against sorafenib when *THY1* gene expression was knocked down in CD90^+^ cell lines *in vitro*. The data suggested that the effect of sorafenib was not directly mediated through the CD90 and its downstream signaling. Therefore, the reduction of CD90^+^ cells might be a by stander effect of sorafenib targeting various signaling pathways including c-Kit. The detailed mechanisms of the functional role of CD90 should be evaluated in future.

Our data suggest that sorafenib is more effective if CD90^+^ CSCs proliferate through the activation of TGF-β signaling. This might be one of the reasons why sorafenib could prolong overall survival without any overt clinical response in patients with advanced HCC^15, 16^. Recent studies suggest that sorafenib treatment might be more beneficial to patients without vascular invasion^25, 26^. However, inhibition of c-Kit signaling alone by imatinib mesylate showed limited efficacy on advanced HCC^27^, suggesting the importance of blocking additional pathways activated in HCC^7^. Since our data suggest that sorafenib had limited effects on tumorigenic EpCAM^+^ CSCs, additional molecularly targeted agents such as HDAC inhibitors or PARP inhibitors may be required to target EpCAM^+^ CSCs in combination with sorafenib^9, 10, 28^.

Recent data indicated that cell-cell communication mediated by EVs plays a fundamental role in tumor development and metastasis^14^. Here, for the first time, we provide novel evidence that sorafenib reduced EVs secretion by CD90^+^ CSCs. Interestingly, EVs containing CD63 mRNA produced by EpCAM^+^ CSCs were increased by sorafenib treatment. One explanation for the reduction of EVs secreted from CD90^+^ CSCs, but not from EpCAM^+^ CSCs, might be the difference in the chemosensitivity of EpCAM^+^ and CD90^+^ CSCs to sorafenib. As sorafenib enriched the population of EpCAM^+^ CSCs, it is possible that CSCs play a central role in the secretion of EVs and affect tumor growth and metastasis in HCC. Sorafenib is highly effective in suppressing EVs containing mRNA encoding *CD63* and *TGFB1* by CD90^+^ CSCs, which may be related to the ability of sorafenib to prolong overall survival in advanced HCC patients and suppress *de novo* metastasis. Our data should help clarify sorafenib’s novel anti-cancer mechanism of inhibiting cellular communication mediated through EVs. Thus, future studies are warranted to explore more effective molecularly targeted agents to suppress EVs produced by heterogeneous liver CSCs.

## Methods

### Cell culture and reagents

Six representative HCC cell lines (HuH1, HuH7, Hep3B, HLE, HLF, and SK-Hep-1) were obtained from the JCRB Cell Bank and ATCC. Milano hcc -1, Milano hcc-2, and Milano hcc-3 clones were established as described previously^13^. All HCC cell lines were cultured in Dulbecco’s modified Eagle’s medium (DMEM) supplemented with 10% fetal bovine serum (FBS). Milano hcc-1, hcc-2, and hcc-3 clones were cultured with Iscove’s modified Dulbecco’s medium supplemented with 10% FBS. Sorafenib tosylate was provided by Bayer (Leverkusen, Germany).

### Cytotoxicity assays

For cytotoxicity assays, single cell suspensions of 2.0 × 10^3^ cells were seeded in 96-well plates, and cell density was evaluated at 48 h after seeding using the Cell Counting Kit-8 (Dojindo Laboratories, Kumamoto, Japan) according to the manufacturer’s instructions.

### Wound healing assays

HuH7 and HLF cells were labeled with the lipophilic fluorescence tracer DiO (indicated as green) or DiD (indicated as blue), and incubated in a μ-Slide 8-well chamber overnight. The silicone inserts were detached and the culture medium was replaced with DMEM containing 10% FBS, including 0.1% dimethyl sulfoxide (DMSO) vehicle (control) or 2.5 μM sorafenib dissolved in DMSO to a final concentration of 0.1% (treatment). Immediately after the medium was changed, the cells were cultured at 37 °C in 5% CO_2_ and time-lapse images were captured for 72 h.

### Karyotype analysis by multicolor FISH

Multicolor FISH analysis of Milano hcc-1, hcc-2, and hcc-3 was performed using multicolor FISH probes (Cambio, Cambridge, UK), Leica DMRA2 system, and CW4000 FISH and CW4000 Karyo software (Chromosome Science Labo, Inc., Sapporo, Japan).

### qRT-PCR analysis

Total RNA was extracted using a High Pure RNA Isolation Kit (Roche Diagnostics K.K., Tokyo, Japan) according to the manufacturer’s instructions. The expression of selected genes was determined in triplicate using the 7900 Sequence Detection System (Applied Biosystems, Foster City, CA). Each sample was normalized relative to β-actin expression. The following probes were used: *THY1*, Hs00998133_m1; *EPCAM*, Hs00174816_m1; *KIT*, Hs00174029_m1; *CD63*, Mm01966817_gl; *TGFB1*, Hs00998133_m1; and *ACTB*, Hs999999903_m1.

### Western blotting

Whole cell lysates were prepared using a radioimmunoprecipitation assay buffer. Mouse polyclonal antibodies to c-Kit (Cell Signaling Technology, Inc., Danvers, MA), mouse polyclonal antibodies to phospho-c-Kit (Cell Signaling Technology, Inc.), and a mouse monoclonal anti-β-actin antibody (Sigma-Aldrich Japan K.K., Tokyo, Japan) were used.

### Immunohistochemical and immunofluorescence analyses

Immunohistochemical analysis was performed using Envision + kits (DAKO, Carpinteria, CA) according to the manufacturer’s instructions. An anti-EpCAM monoclonal antibody VU-1D9 (Oncogene Research Products, San Diego, CA) and anti-CD90 antibody 5E10 (STEMCELL Technologies, Vancouver, BC) were used for detecting EpCAM and CD90, respectively, in subcutaneous tumors derived from Milano hcc-1, hcc-2, and hcc-3 as well as original primary HCC tissues.

### FACS analyses

Cultured cells were trypsinized, washed, and resuspended in Hank’s balanced salt solution (Lonza, Basel, Switzerland) supplemented with 1% HEPES and 2% FBS. The cells were then incubated with fluorescein isothiocyanate (FITC)-conjugated anti-EpCAM monoclonal antibody Clone Ber-EP4 (DAKO) and FITC-conjugated anti-CD90 (STEMCELL Technologies) on ice for 30 min, and analyzed using a FACSCalibur (BD Biosciences, San Jose, CA).

### Animal studies

Six-week-old NOD/SCID mice were purchased from Charles River Laboratories, Inc. (Wilmington, MA). The protocol was approved by the Kanazawa University Animal Care and Use Committee, and all methods were performed in accordance with the guidelines and regulations determined by Kanazawa University. A total of 1.0 × 10^6^ tumor cells (a mixture of 5.0 × 10^5^ HuH7 cells and 5.0 × 10^5^ HLF cells) were suspended in 200 μL DMEM and Matrigel (1:1), and then injected subcutaneously into the flank. The incidence and size of subcutaneous tumors were recorded. Sorafenib (treatment) or vehicle (control) was orally administered 3 times per week at 20 days after injection for 2 weeks. For histologic evaluation, formalin-fixed, paraffin-embedded tumor tissue sections were stained with hematoxylin and eosin.,

### Isolation of EVs

A total of 2.0 × 10^5^ HCC cells were harvested from 6-well dishes in 2 mL of 10% FBS DMEM supplemented with control DMSO (0.1%) or sorafenib (2.5 μM). Exosome isolation was performed using ExoQuick TC Tissue Culture Media Exosome Precipitation Solution (System Biosciences, Inc., Palo Alto, CA), according to the manufacturer’s protocol. Briefly, 5 mL culture supernatant were mixed with 1 mL ExoQuick TC Tissue Culture Media Exosome Precipitation Solution (System Biosciences, Inc.). Samples were incubated at 4 °C overnight and centrifuged at 1500 rpm for 30 min. The protein-rich supernatant was removed and the exosome-rich pellet was used directly for RNA extraction and subsequent qRT-PCR analysis in triplicate.

### Visualization of EV kinetics

HuH7 and HLF cells were stained with DiO (Sigma-Aldrich Japan K.K.) and PKH-26 (Sigma-Aldrich Japan K.K.) for visualization of cellular lipids and EVs, respectively^29^. The cells were co-cultured for 72 h with control DMSO (0.1%) or sorafenib (2.5 μM).

### Statistical analyses

Student’s *t* test was used to compare various test groups assayed by quantitative qRT-PCR analysis and cell proliferation assays. χ^2^ tests were used to evaluate the frequency of lung metastasis treated with vehicle (control) or sorafenib *in vivo*. All analyses were performed using GraphPad Prism software (La Jolla, CA).

## Electronic supplementary material

Supplementary data

Supplementary movie 1

Supplementary movie 2

Supplementary movie 3
