# Supplementary material for: Sorafenib suppresses extrahepatic metastasis de novo in hepatocellular carcinoma through inhibition of mesenchymal cancer stem cells characterized by the expression of CD90
Source: Sci Rep. 2017 Sep 12;7:11292. doi: 10.1038/s41598-017-11848-z (PMC5596021; doi:10.1038/s41598-017-11848-z)
Supplement: Supplementary file 1 — Supplementary data [file 41598_2017_11848_MOESM1_ESM.pdf]

Title: Sorafenib suppresses extrahepatic metastasis *de novo* in hepatocellular carcinoma through inhibition of mesenchymal cancer stem cells characterized by the expression of CD90

Mariko Yoshida[1], Taro Yamashita[1, 2] *, Hikari Okada[1], Naoki Oishi[1], Kouki Nio[1], Takehiro Hayashi[1], Yoshimoto Nomura[1], Tomoyuki Hayashi[1], Yoshiro Asahina[1], Mika Ohwada[1], Hajime Sunagozaka[1], Hajime Takatori[1], Federico Colombo[3], Laura Porretti[3], Masao Honda[1], and Shuichi Kaneko[1]

Supplementary Information

Supplementary Figure Legends

Supplementary Figure 1. Effect of EpCAM/CD90 knockdown on cell proliferation. **A and B**. qRT-PCR analysis of *EPCAM* or *THY1* expression in HuH7 or HLF cells. Si-RNAs targeting control (SI03650318; QIAGEN), *EPCAM* (Silencer Select S 8370 and S8371; Ambion), or *THY1* (Silencer Select s14126 and s14127; Ambion) were transfected using Lipofectamine 2000. Gene expression was evaluated at 48 h after transfection in triplicate. **C and D**. Cell proliferation assay of HuH7 or HLF cells treated with si-RNAs. Cell density was measured at 72 h after transfection in quadruplicate.

Supplementary Figure 2. Tumorigenicity of Milano hcc-2. **A**. FACS analysis of Milano hcc-2 cells. Pure EpCAM$^+$ or CD90$^+$ cells were isolated using a BD FACSAriaII cell sorter, and greater than 98.5% purity was confirmed in isolated cells using FACSCalibur. These

cells were cultured for 30 days, and the expression of EpCAM and CD90 was evaluated using FACSCalibur. **B and C.** Tumorigenic capacity of 5.0 × 10^5 $EpCAM^+$ Milano hcc-2 cells and 5.0 × 10^5 unsorted Milano hcc-2 cells injected into a subcutaneous lesion. Sorted $EpCAM^+$ cells had higher tumorigenic capacity compared with the unsorted cells, but the difference did not reach statistical significance (P = 0.18).

Supplementary Fig. 3. A. Chemosensitivity of sorted $EpCAM^{+/-}$ HuH7 cells to sorafenib. EpCAM+/- cells were isolated using a FACSAriaII cell sorter and incubated in 96-well plates overnight. The cells were then treated with 5 μM sorafenib for 72 h and cell density was measured in quadruplicate. **B.** Chemosensitivity of Huh7 cells to sorafenib by *EPCAM* knockdown. Control or EpCAM-targeted si-RNAs were transfected in Huh7 cells and then treated with 2.5 μM sorafenib for 48 h and cell density was measured in quintuplicate. **C.** Chemosensitivity of HLF cells to sorafenib by *Thy1* knockdown. Control or Thy1-targeted si-RNAs were transfected in HLF cells and then treated with 2.5 μM sorafenib for 48 h and cell density was measured in quintuplicate.

Supplementary Fig. 4. Effect of sorafenib on Milano hcc-2. **A.** Tumorigenic capacity of 5.0 × 10^5 Milano hcc-2 cells injected into a subcutaneous lesion and treated with vehicle or sorafenib. Sorafenib (30 mg/kg/day, 100 μL/mice, n = 8) or vehicle (100 μL/mice, n = 8) was orally administered 3 times per week at 30 days after injection for 2 weeks (day 30 to 44). **B.** Microscopic analysis of metastasis in the lung of NOD/SCID mice treated with vehicle or sorafenib. **C.** Frequency of lung metastasis in the lung of NOD/SCID mice treated with vehicle (n = 8) or sorafenib (n = 8).

Supplementary Movie 1. EpCAM$^+$ Huh7 cells (green) and CD90$^+$ HLF cells (red) was mixed well and dispersed equally in the well and co-cultured for 72 h using a CSU-X1 spinning disk (Yokogawa) and Andor iXon3 EMCCD camera system (Andor Technology). Images were analyzed by iQ Software (Andor Technology).

Supplementary Movies 2 and 3. A wound healing assay was performed to evaluate cell motility using μ-Slide 8-well chambers. Cell motility of HuH7 cells (green) co-cultured with HLF cells (blue) treated with control (0.1% DMSO) (Supplementary Movie 1) or sorafenib (5 μM) (Supplementary Movie 2) was monitored in real-time by time-lapse image analysis. The cells were cultured at 37°C in 5% CO_2 and time-lapse images were captured for 72 h using a CSU-X1 spinning disk (Yokogawa) and Andor iXon3 EMCCD camera system (Andor Technology). Images were analyzed by iQ Software (Andor Technology).

Supplementary Fig. 1

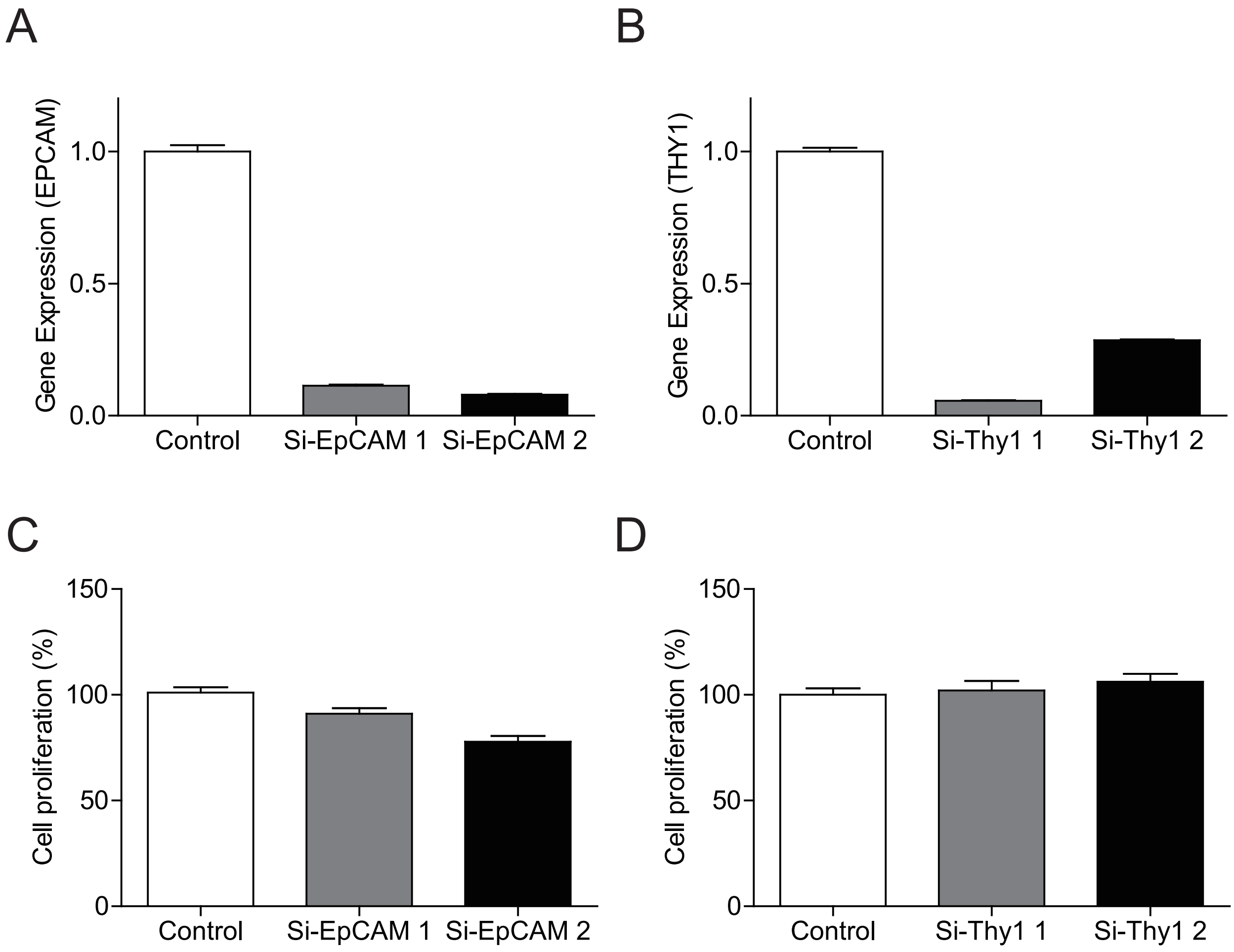

Supplementary Fig. 2

A

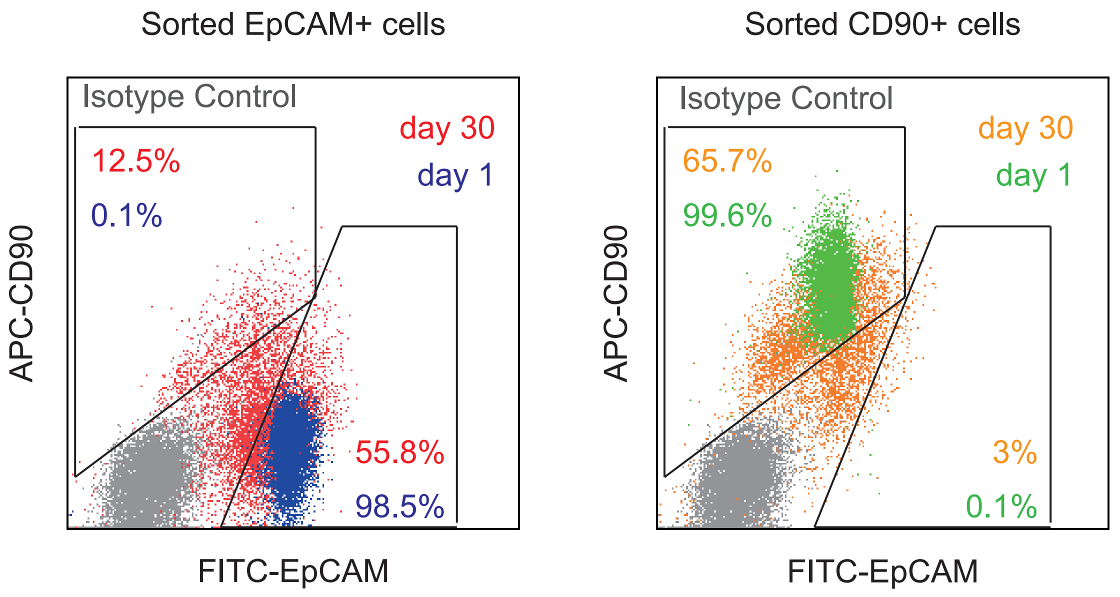

B

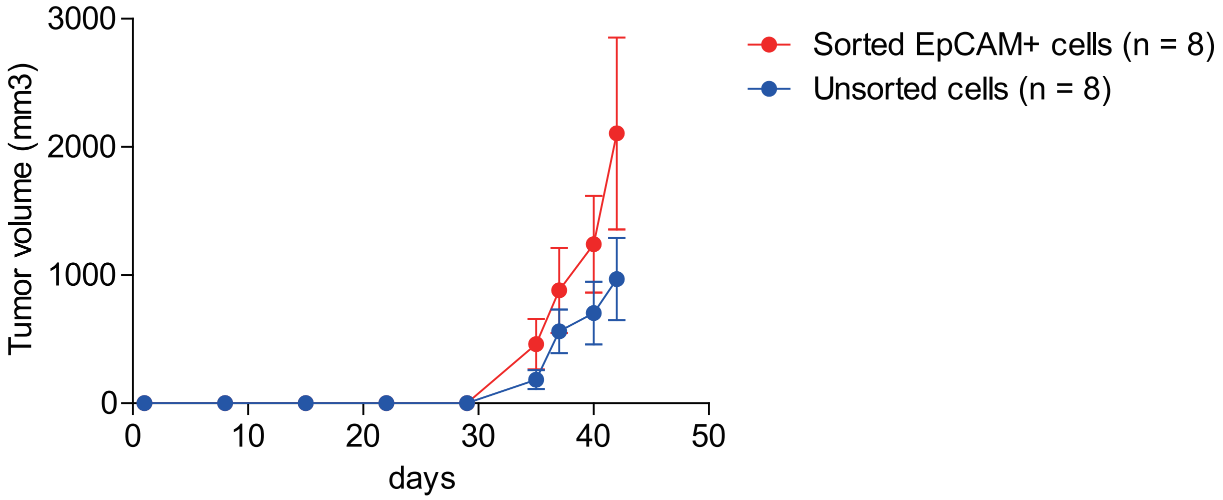

C

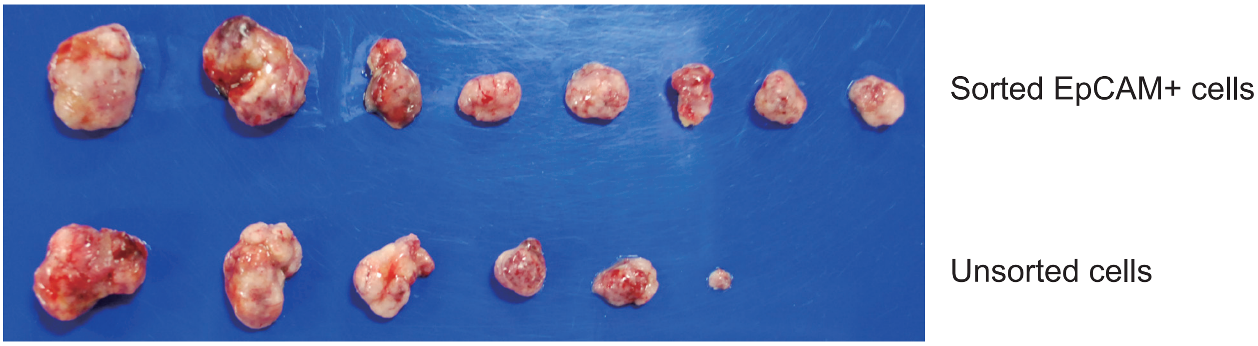

Supplementary Fig. 3

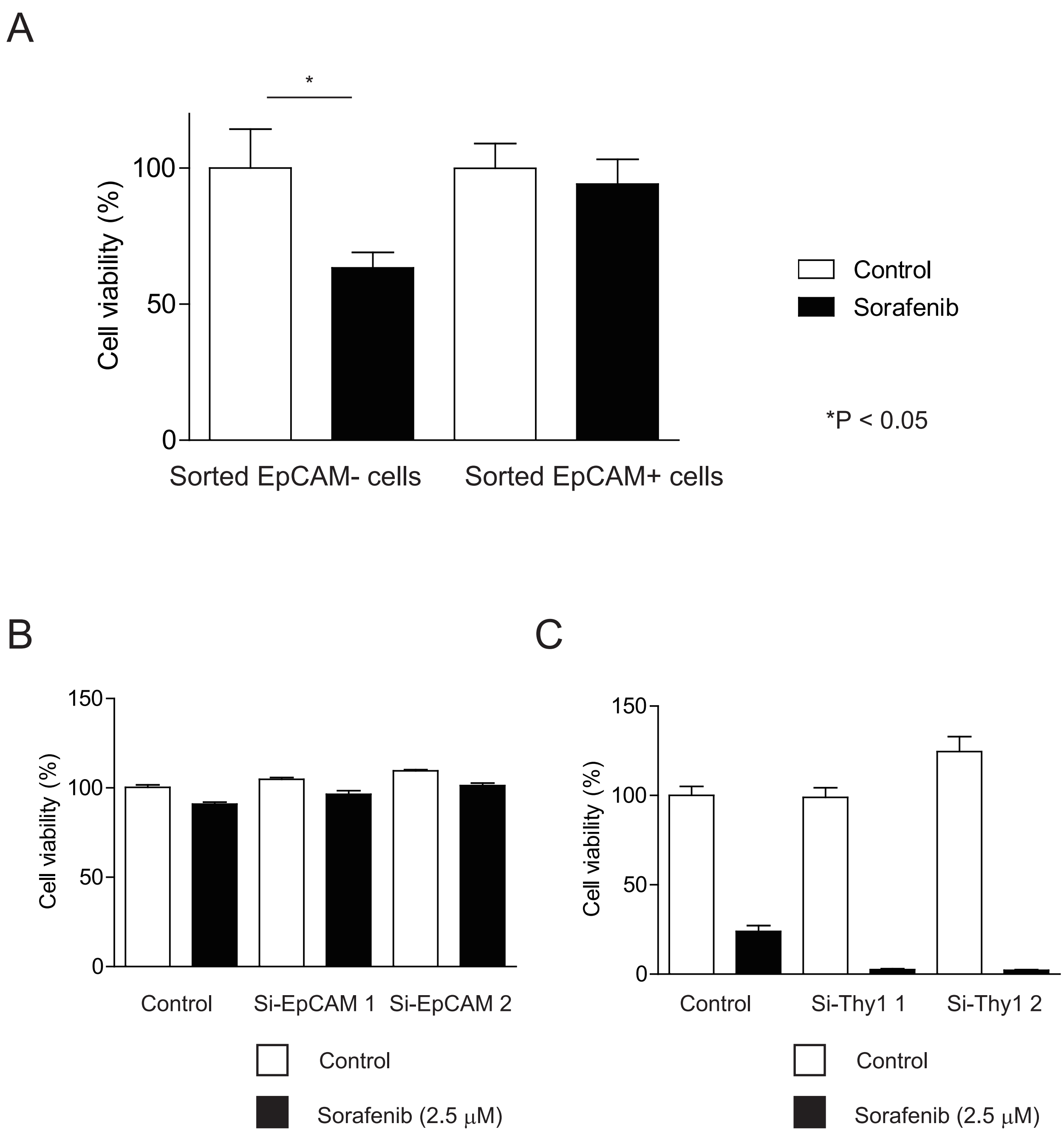

Supplementary Fig. 4

A

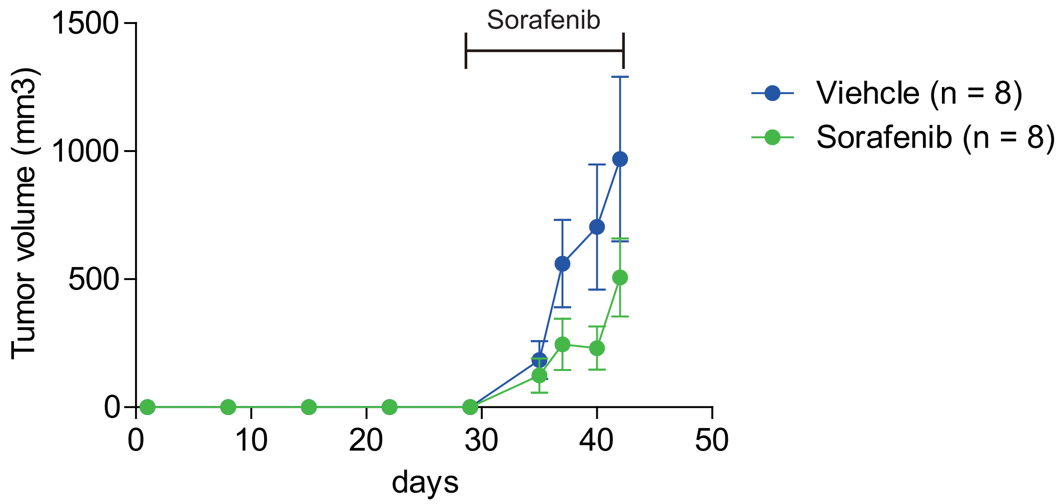

B

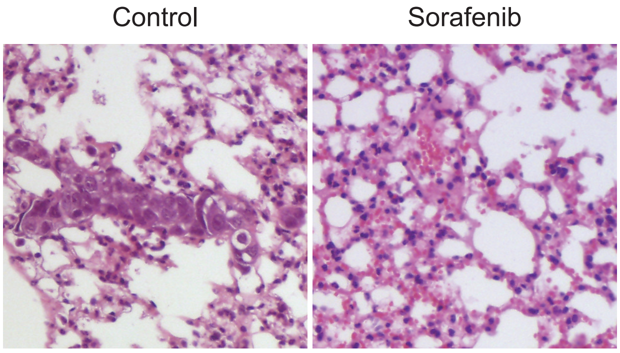

C

Lung metastasis	Yes	No
Control	5	3
Sorafenib	0	8

P = 0.025
